# A machine learning–based triage system for systemic EBV-positive T/NK cell lymphoproliferative diseases of childhood

**DOI:** 10.1172/jci.insight.180837

**Published:** 2026-02-23

**Authors:** Pujun Guan, Zihang Chen, Hanze Dong, Xia Guo, Juan Huang, Tian Dong, Mi Wang, Xiaoxi Lu, Fei Huang, Wenbin Li, Yuan Tang, Li Zhang, Ling Pan, Ju Gao, Shikun Wang, Rongbo Liu, Wenyan Zhang, Sha Zhao, Weiping Liu

**Affiliations:** 1 Department of Pathology and; 2 Department of Radiology, West China Hospital, Sichuan University, Chengdu, China.; 3 Department of Mathematics, The Hong Kong University of Science and Technology, Hong Kong, China.; 4 Department of Pediatrics, West China Second University Hospital, Sichuan University, Chengdu, China.; 5 Department of Pathology, The Affiliated Hospital of Southwest Medical University, Luzhou, China.; 6 Department of Hematology and; 7 Department of Dermatology, West China Hospital, Sichuan University, Chengdu, China.; 8 MOE Laboratory of Biosystem Homeostasis and Protection and Life Sciences Institute, Zhejiang University, Hangzhou, China.; 9 Department of Biostatistics, Columbia University Medical Center, New York, New York, USA.

**Keywords:** Hematology, Infectious disease, Lymphomas

## Abstract

Systemic Epstein-Barr virus–positive (EBV-positive) T/NK cell lymphoproliferative diseases of childhood (sEBV^+^T/NK-LPD) are a spectrum of rare diseases that have highly variable biological behavior, from indolent conditions to highly aggressive malignancies. Clinicians currently face substantial challenges in promptly assessing disease severity and predicting patient outcomes, leading to limitations in treatment planning. To address this challenge, we constructed a comprehensive triage system to aid in rapid clinical interventions. The study included 156 patients with newly diagnosed sEBV^+^T/NK-LPD from 42 institutions. An independent prospective cohort of 35 newly enrolled patients was further included to evaluate the model’s performance. An additional 45 patients from the literature and 18 patients who underwent hematopoietic stem cell transplantation were included to test the score’s generalizability. An integrative machine learning strategy was applied to identify robust and optimal factors and to integrate multiple algorithms to enhance the system’s performance and stability. This system, termed COLLAPSED, identifies critical factors and provides a stable, high-performing ensemble. This model was validated externally and simplified into a risk score to improve interpretability and accessibility. The COLLAPSED system substantially enhances clinicians’ ability to rapidly and precisely identify high-risk patients, thus enabling timely clinical decision-making and expedited initiation of potentially lifesaving treatments.

## Abstract 2

The COLLAPSED system is the first ML-based triage system that was developed and validated for sEBV+T/NK-LPD through small-data-friendly machine-learning techniques.

## Introduction

The systemic Epstein-Barr virus–positive (EBV-positive) T/NK cell lymphoproliferative diseases (or disorders) of childhood (sEBV^+^T/NK-LPD), also known as systemic EBV-positive T cell and NK cell lymphoid proliferations and lymphomas of childhood, comprise a spectrum of rare conditions mainly affecting Asian and Hispanic children and young adults (1). These diseases include systemic EBV-positive T cell lymphoma of childhood (STLC) and 2 systemic forms of chronic active EBV disease (CAEBVD): systemic chronic active EBV disease (sCAEBVD) and systemic hydroa vacciniforme lymphoproliferative disorder (sHV-LPD). All these diseases originate from EBV-positive T cells and/or NK cells, often manifesting as monoclonal proliferation. They can all present with systemic symptoms, including multiple lymphadenopathies and elevated EBV-DNA levels in peripheral blood. Multiple extranodal organs, such as the spleen, liver, skin, and bone marrow, can be involved, frequently accompanied by hemophagocytic lymphohistiocytosis. Additionally, the systemic forms of CAEBVD may transform into STLC. While STLC typically follows a fulminant clinical course, the prognosis for CAEBVDs varies. Accurately diagnosing subclasses within sEBV^+^T/NK-LPD poses considerable challenges for even experienced hematopathologists, particularly in the early stages, and precise diagnosis can be delayed (2, 3).

The substantial heterogeneity within sEBV^+^T/NK-LPD poses considerable difficulties for clinicians assessing disease aggressiveness and predicting patient outcomes. Patients with similar clinical and pathological features may experience markedly different prognoses (4–6). Therefore, an early and comprehensive risk assessment is essential for effective patient management and informed treatment planning. Allogeneic hematopoietic stem cell transplantation (HSCT) remains the most effective therapeutic option for sEBV^+^T/NK-LPD, yet its preparatory steps, such as eligibility evaluation and donor identification, often require a substantial amount of time. For instance, one study reported a median interval of 19 months from diagnosis to HSCT among patients with various hematological diseases, with over 40% of patients waiting more than 6 months (7). Given the rapidly progressive nature of sEBV^+^T/NK-LPD, wherein nearly half of patients die within 8 months despite antitumor therapies, it is critical to promptly identify patients who require immediate intervention and expedite their evaluation for HSCT, even if initial clinical and pathological presentations are broad or nonspecific.

Although several factors linked to patient risk have emerged, variations in reports persist (4, 6, 8). All of these factors have yet to be comprehensively evaluated and externally validated. Consequently, risk-prediction models for sEBV^+^T/N-LPD are currently absent in clinical environments. Moreover, the potential insights from radiological features have been underexplored. Crucially, integrating clinical, laboratory, pathological, and radiological data to enhance risk prediction remains challenging. This integration is vital for more effective patient triage, paving the way for personalized management, optimal allocation of clinical resources, and identifying innovative therapies.

The medical community has been recognizing the importance of tools powered by machine learning (ML) in the era of precision medicine (9). ML has been successfully applied to common diseases, which benefit from the availability of extensive datasets. However, when it comes to rare diseases or high-dimensional data, such as data regarding sEBV^+^T/NK-LPD, challenges arise because of the vast number of variables relative to the limited number of patients. Despite these hurdles, recent research sheds light on the promise of ML algorithms, suggesting their capability to create practical clinical tools even for rare diseases (10).

**Figure 1:**
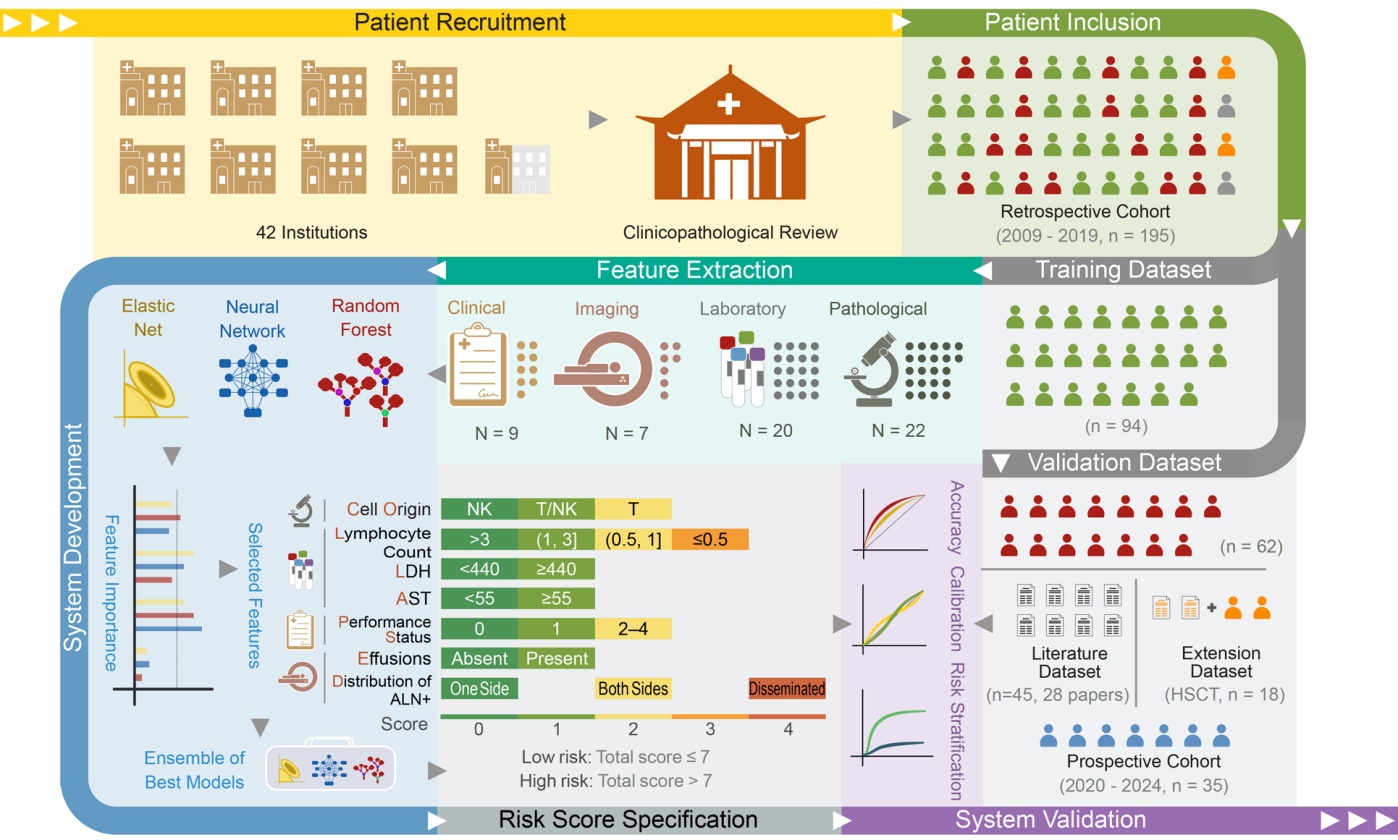
Overview of study design and COLLAPSED system development. Suspected cases from 42 institutions were screened and reviewed according to the diagnostic criteria of the WHO classification. Eligible patients were randomly split into a training dataset (*n* = 94) and an internal validation dataset (*n* = 62). A total of 58 clinical, imaging, laboratory, and pathological features were collected from which 7 robust predictors were selected through an integrative feature selection strategy. These features were used to develop an ensemble machine learning model (COLLAPSED) for risk prediction, and a simplified COLLAPSED score was derived for clinical risk stratification. Model accuracy, calibration, and risk-stratification performance were evaluated using the internal validation dataset, and further assessed using an external literature cohort (*n* = 45), an extension cohort (*n* = 18, patients who received hematopoietic stem cell transplantation [HSCT]), and a prospective cohort (*n* = 35).

To tackle this problem, we constructed an ML-based triage system for patients with sEBV^+^T/NK-LPD by strategically utilizing high-dimensional-data-friendly ML tools (Figure 1). The system was validated in multiple independent patient sets and was found to use factors consistent with prior knowledge. This system will streamline risk assessment in patients with sEBV^+^T/NK-LPD and lay a foundation for developing clinical tools tailored to rare diseases.

## Results

### Patient characteristics.

The clinicopathological features of patients were comparable between the training dataset (*n* = 94) and the validation dataset (*n* = 62) (Supplemental Table 1; supplemental material available online with this article; https://doi.org/10.1172/jci.insight.180837DS1). The overall survival in both datasets was also similar: 72 patients (76.6%) died of the disease in the training dataset (median survival: 8 [95% CI, 6–13] months), and 44 patients (71.0%) died in the validation dataset (median survival: 7 [95% CI, 4–17] months). The median follow-up was 30 (IQR, 14–46) months and 27 (IQR, 16–33) months in the training and validation datasets, respectively. In the prospective validation cohort, 16 patients died of the disease. The median survival of the cohort was 44 (95% CI, 13–NA) months, and the median follow-up was 21 (IQR, 16–31) months (Supplemental Table 2).

### Selected features.

**Figure 2:**
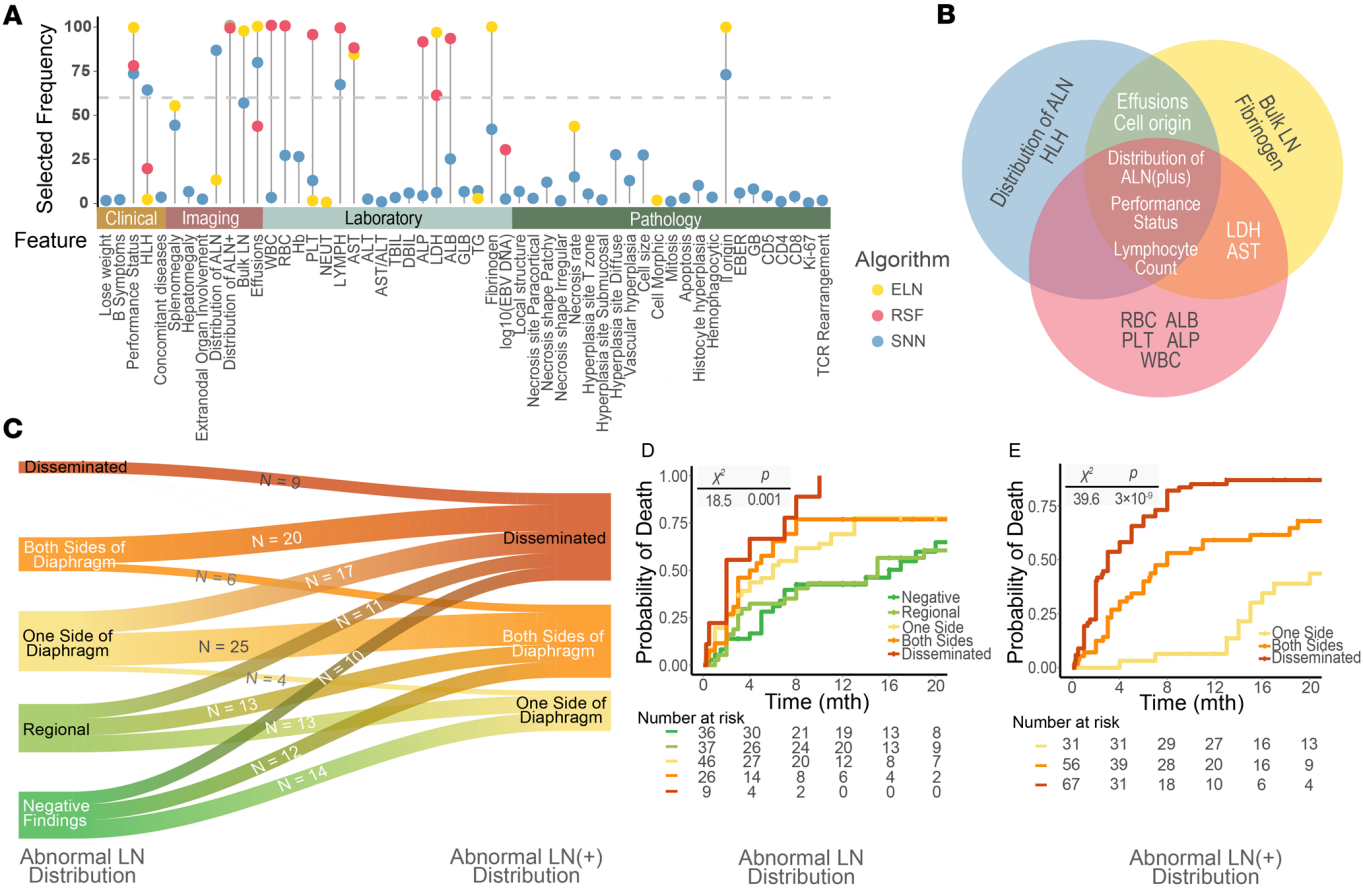
Feature selection in the COLLAPSED system and LN distribution analysis. (**A**) Frequencies with which each candidate feature appeared among the top 10 most influential variables across 2,100 model runs using 3 algorithms: elastic net (ELN), random survival forest (RSF), and survival neural network (SNN). The dashed line denotes the 60% selection-frequency threshold. (**B**) Venn diagram summarizing features selected by the 3 base algorithms. Distribution of ALN+, performance status, and lymphocyte counts were identified by all 3 algorithms; LDH and AST by ELN and RSF; effusion and cell origin by SNN and ELN. ALB, albumin; ALP, alkaline phosphatase; HLH, hemophagocytic lymphohistiocytosis; PLT, platelets. (**C**) Alluvial plot illustrating reclassification of patients according to 2 abnormal-lymph-node definitions (ALN vs. ALN+). Curve width corresponds to the number of patients in each transition between categories. (**D**) Risk stratification based on ALN distribution. (**E**) Risk stratification based on ALN+ distribution. Group differences in **D** and **E** were compared using the log-rank test.

A total of 58 candidate features were included, consisting of 9 demographic and clinical features, 7 imaging features, 20 laboratory features, and 22 pathological features (Supplemental Table 1). Through the robust feature selection process, 7 features were selected for the model, “COLLAPSED”: cell origin, lymphocyte counts, lactate dehydrogenase (LDH), aspartate aminotransferase (AST), performance status (PS), effusion, distribution of abnormal lymph nodes (plus) (distribution of ALN+) (see Supplemental Table 3 for details). In comparison with any single feature selection approach, the number of selected features is considerably reduced after repetition of the selection process and integration of the results from multiple base algorithms (Figure 2, A and B). These results support that the selection algorithm with a resampling-based method, multiple initializations, and an ensemble approach substantially enhances the robustness of the feature selection.

The distribution of ALN was assessed using the Lugano classification, whereas the distribution of ALN+ also considered LN groups that showed a notable increase in the number of LNs, regardless of their size. From the entire dataset, 36 patients had negative findings, and 37 displayed regional involvement of regional LN groups per ALN criteria. These patients exhibited increased LNs across at least 2 LN groups and were reclassified. Specifically, approximately one-third had abnormal LN groups on 1 side of the diaphragm, another third had them on both sides, and the last third showed a disseminated pattern per ALN+ criteria. Among those with ALN groups detected on more than 1 side of the diaphragm, the majority were up-classified based on ALN+ criteria until reaching the highest classification (Figure 2C). In evaluation of the risk of death, the distribution of ALN revealed overlapping risk curves for patients with negative findings and those exhibiting regional LN group involvement. Similarly, overlaps were observed between patients with ALN present on both sides of the diaphragm and those displaying a disseminated pattern after 1 year (Figure 2D). In contrast, when the ALN+ criteria were used, the risk curves were distinctly separated (Figure 2E). We presented exemplary CT images highlighting small LN increases in various anatomical regions (Supplemental Figure 1A). Furthermore, a biopsy confirmed disease involvement in a small LN (Supplemental Figure 1B).

### Model performance.

**Figure 3:**
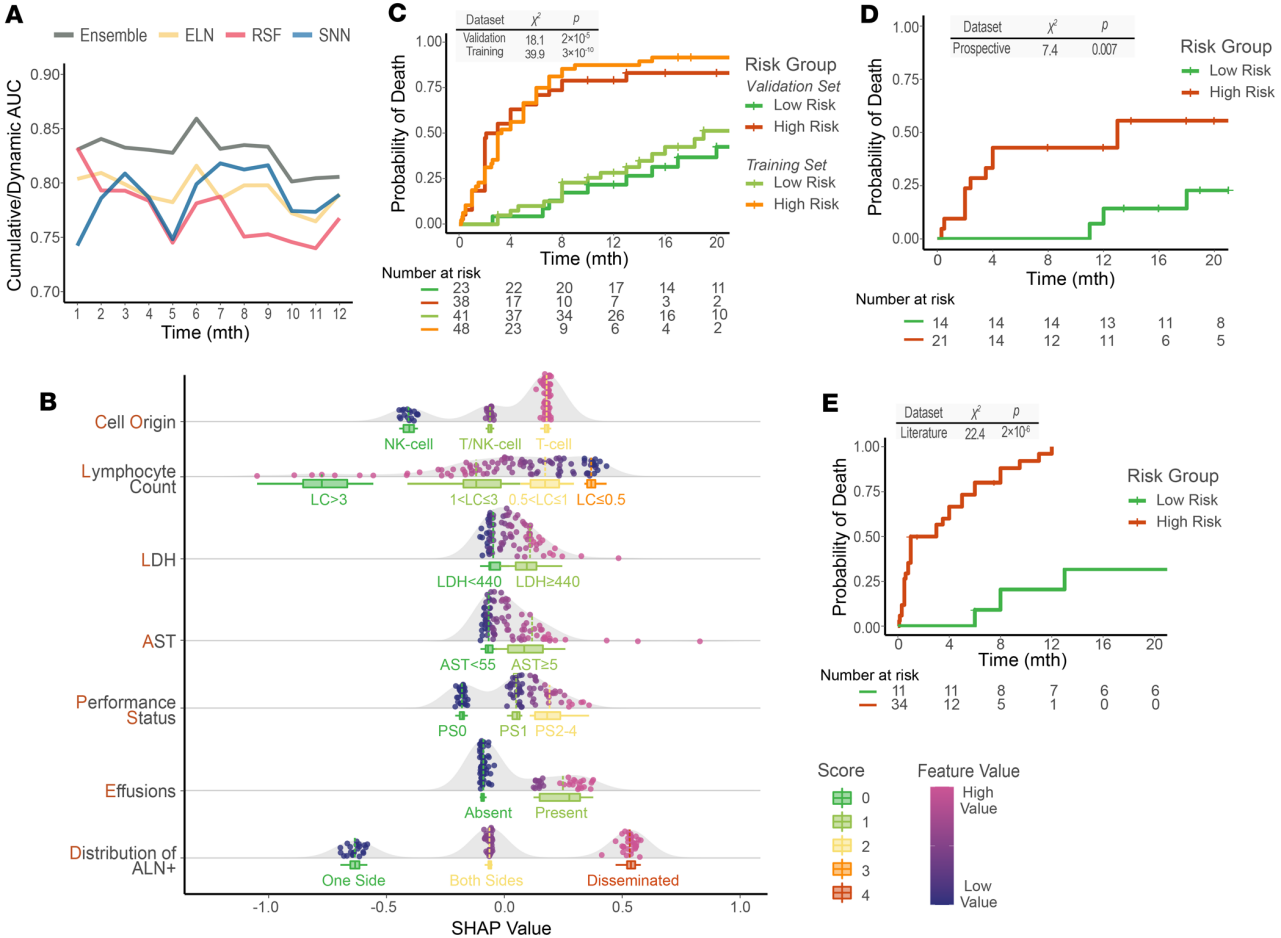
Evaluation of the COLLAPSED system across cohorts. (**A**) Time-dependent discriminative performance of the ensemble model and its 3 base algorithms (ELN, RSF, and SNN) over time, evaluated by the cumulative/dynamic area under the time-dependent receiver operating characteristic curve (AUC). (**B**) Distribution of feature importance (SHAP values) and binned scores of the 7 selected predictors within the training dataset. The color gradient from dark blue to light purple represents the relative feature values. Box plot colors correspond to score bins (0 to 4) assigned to each predictor, and the dashed line marks the mean SHAP value within each bin. LC, lymphocyte count. (**C**) Risk stratification based on the COLLAPSED score in the training and validation datasets (the primary retrospective cohort). (**D**) Risk stratification based on the COLLAPSED score in the prospective cohort. (**E**) Risk stratification based on the COLLAPSED score using the literature-derived cohort. Group differences in **C**–**E** were compared using the log-rank test.

The final COLLAPSED model was obtained by averaging of the best model of each base algorithm in the internal verification process. To benchmark the final model (ensemble model), we compared it with the best model of each base algorithm — elastic net (ELN), random survival forest (RSF), and survival neural network (SNN) — which were generated during the feature selection. For the validation dataset, the 6-month C indices were 0.798 (95% CI, 0.704–0.884) for the ensemble model, 0.754 (95% CI, 0.652–0.845) for the ELN model, 0.729 (95% CI, 0.622–0.827) for the RSF model, and 0.720 (95% CI, 0.612–0.818) for the SNN model. The 1-year C indices were 0.773 (95% CI, 0.683–0.857) for the ensemble model, 0.738 (95% CI, 0.642–0.826) for the ELN model, 0.708 (95% CI, 0.612–0.797) for the RSF model, and 0.726 (95% CI, 0.639–0.811) for the SNN model. The ensemble model outperformed the others in the cumulative/dynamic AUC analysis, with calibration plots showing comparable calibration for most models (Figure 3A and Supplemental Figure 2A). Further quantification can be found in Supplemental Table 4. This comparison highlights the superior performance of the ensemble model, even with fewer features than the individually constructed ELN, RSF, and SNN models.

To evaluate temporal generalizability, the model was further tested in a prospective cohort of 35 newly enrolled patients (2020 to 2024). Despite differences in the collection period, the model maintained robust discriminative ability, with 6-month and 1-year C indices of 0.906 (95% CI, 0.819–0.981) and 0.781 (95% CI, 0.533–0.959), respectively.

### Model interpretation and risk score development.

To understand how the COLLAPSED model utilizes the features, we calculated the Shapley additive explanations (SHAP) values to quantify the contribution of each feature to the model output (Figure 3B). This analysis revealed that sEBV^+^T/NK-LPD patients with T cell origin, lymphocytopenia, liver dysfunction, compromised PS, serous cavity effusions, and disseminated disease have an increased risk. The alignment of these findings with well-established medical knowledge and clinical observations reinforces our confidence in the model’s accuracy and relevance.

The COLLAPSED score was derived from the COLLAPSED model to make triaging of patients more convenient. The distribution of SHAP values and the corresponding score are depicted in Figure 3B and Supplemental Table 3. The total COLLAPSED score was highly correlated with the predictors from the ensemble model (Supplemental Figure 2B; Spearman’s rank correlation ρ = 0.963, *P* < 10^–15^), demonstrating its usefulness as an approximation of the patient’s risks.

### Risk group specification.

Patients were classified into low-risk (total score ≤ 7) and high-risk (score > 7) groups. The median survival was 26 (95% CI, 17–NA) months for the low-risk group and 3 (95% CI, 2–6) months for the high-risk group in the validation dataset, which mirrored the results in the training dataset (low-risk vs. high-risk: 19 [95% CI, 15–37] vs. 3 [95% CI, 3–5] months). The risk of death from the disease between the 2 groups was significantly different (Figure 3C): The estimated 6-month risk in the validation dataset was 4.3% (95% CI, 0.0%–12.3%) for the low-risk group and 71.1% (95% CI, 52.4%–82.4%) for the high-risk group, which was also consistent with the results from the training dataset (low-risk vs. high-risk: 10.0% [95% CI, 0.0%–18.9%] vs. 75.0% [95% CI, 59.2%–85.7%]).

In the prospective validation cohort, patients were similarly stratified. The median survival was 13 months (95% CI, 3.5–NA) for the high-risk group, whereas the median survival for the low-risk group was not reached. At 6 months, the estimated risk of death was 42.9% (95% CI, 17.2%–60.5%) for the high-risk group, whereas deaths in the low-risk group had not yet occurred, consistent with the risk separation seen in the retrospective cohorts (Figure 3D).

To assess the generalizability of the COLLAPSED score, a literature dataset consisting of 45 patients was curated. The median follow-up was 36 (IQR, 25–48) months. The median survival for the high-risk group was 2 (95% CI, 1–5) months, while the median survival for the low-risk group was not reached. The estimated 6-month risk of death was 80.0% (95% CI, 59.5%–90.1%) for the high-risk group and 9.1% (95% CI, 0%–24.6%) for the low-risk group (Figure 3E).

### Subgroup analysis and extension.

**Figure 4:**
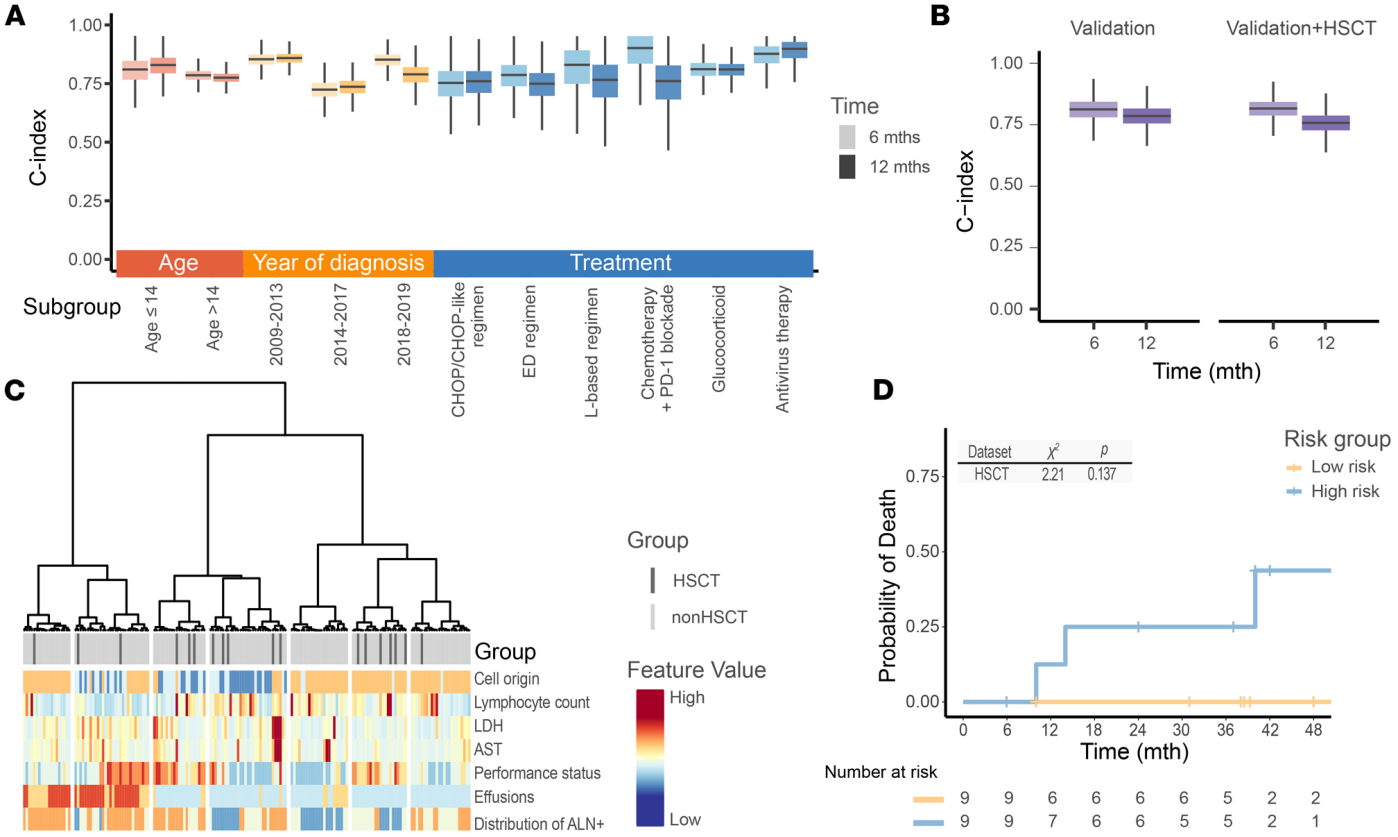
Subgroup analysis and evaluation of model generalizability. (**A**) Model performance across patient subgroups, evaluated by the bootstrapped C index at 6 (light color) and 12 (dark color) months. Box plots show the median and interquartile range (IQR), with whiskers extending to 1.5 × IQR. (**B**) Model performance on the validation dataset and on the combined validation plus HSCT datasets, evaluated by the bootstrapped C index at 6 (light color) and 12 (dark color) months. (**C**) Hierarchical clustering of patients based on Spearman’s correlations among the selected feature values. The heatmap uses a blue-to-red gradient to represent relative feature values; branch lengths indicate patient dissimilarity. (**D**) Risk stratification of patients with HSCT according to the COLLAPSED score. Group differences were assessed using the log-rank test.

Additionally, the discrimination capability of the COLLAPSED system was assessed in patient subgroups defined by age, year of diagnosis, and type of treatment. The results showed that the C indices in all subgroups were greater than 0.77, suggesting the system’s robust performance (Figure 4A and Supplemental Table 5).

The C index values were comparable for both the standalone validation dataset and the validation dataset combined with the hematopoietic stem cell transplantation (HSCT) dataset (extension dataset; Figure 4B). Hierarchical clustering of patients with or without HSCT revealed no evidence of bias in selecting HSCT based on any features chosen in the COLLAPSED system. Among the patients without HSCT, those closest to the ones receiving HSCT displayed comparable baseline characteristics (Figure 4C). Among those designated as high-risk and undergoing HSCT, only 3 patients died, whereas none from the low-risk group did (Figure 4D). Although this difference did not reach statistical significance owing to the limited sample size, it might suggest a potential trend toward worse outcomes for high-risk patients even after HSCT, indicating the system’s potential compatibility with datasets incorporating HSCT patients.

## Discussion

This study introduces, to our knowledge, the first triage system for sEBV^+^T/NK-LPD, demonstrating its robust performance through external validation. Before our research, clinicians primarily relied on a qualitative description of risk factors rather than a quantitative assessment (11). Our ML-powered triage system offers a standardized, quantitative approach that systematically integrates these diverse risk factors, building on and refining the collective clinical intuition. The COLLAPSED system is well suited for clinical applications. Testing on multiple independent datasets has demonstrated its accuracy and robustness. A simple risk score derived from the model demystifies the inherent “black box” of complicated ML algorithms and transmutes it into a concise, interpretable metric (Figure 1). To fortify accessibility, an online calculator (https://www.ebvlpd.org.cn/collapsed) and a checklist (Supplemental Table 3) are provided.

The features selected for the system reflect the intrinsic biological character and dissemination pattern of disease and its impact on the human body, particularly on the hematopoietic, metabolic, and immune systems. Several features were demonstrated to have prognostic value in sEBV^+^T/NK-LPD or other lymphomas (4, 12, 13). The distribution of ALN+ was selected 100 times by all base algorithms and showed superior discrimination ability over the Lugano classification, which underscores its effectiveness as a refined adaptation of the Lugano classification, specifically tailored for sEBV^+^T/NK-LPD. The update captures the dualistic nature of sEBV^+^T/NK-LPD, which manifests both tumor and infectious characteristics, by considering LN enlargement and the increase in smaller LNs (14). ALN+ can be instrumental for radiologists, directing their focus toward these “dangerous small LNs” and ensuring that such subtle yet critical abnormalities do not go unnoticed in clinical evaluations.

The COLLAPSED system aids in identifying blind spots within clinical experience. With optimal weighting of 7 robust features, it identifies 2 categories of high-risk patients who might previously have been overlooked. One group of patients is in the early stage of an episode, exhibiting only mild abnormalities in their risk factors. Each individual abnormality may not be pronounced enough to capture a doctor’s immediate attention, but most identified risk factors are affected. Another category of patients demonstrates a temporary tolerance to disease dissemination. Despite the presence of increased small LNs throughout their body, they maintain a relatively stable general health condition for a short period. Often, they exhibit no dramatic shifts in liver and hematopoietic function at first. Both types of patients are at risk of rapid progression and have an increased risk of death within 6 months. Early detection of these at-risk patients is crucial to prevent delayed treatment and improve overall care.

Furthermore, the COLLAPSED system offers potential benefits in treatment planning. Currently, various therapies have been employed, ranging from antiviral therapy and glucocorticoids to different chemotherapy regimens and immunotherapies (4, 6, 15, 16). While the effectiveness of these treatments is often uncertain, HSCT has demonstrated substantial improvements in survival outcomes, particularly when the timing of transplantation is optimized (11). High-risk patients, whose 6-month survival rate is below 30%, should promptly receive chemotherapy and/or immunotherapy to maximize their chances of proceeding to immediate HSCT. Conversely, for low-risk patients, the optimal timing for HSCT is during the inactive phase of the disease to enhance long-term survival (Supplemental Figure 3). However, it is important to acknowledge that the current perspectives and proposed strategies are largely based on empirical considerations, primarily driven by the very limited therapeutic window. Therefore, prospective studies are urgently needed to better define optimal treatment strategies for each risk category. Patients might benefit from participation in well-designed clinical trials. The development of alternative treatments remains essential owing to the inherent mortality risks of HSCT and the necessity to provide effective treatments for patients who are ineligible for transplantation. Additionally, medical strategy planners and policymakers should prioritize high-risk patients by incorporating them into expedited or “green channel” admission processes, thereby ensuring timely access to maximize their chance for HSCT.

The modeling technique has some advantages in datasets generated from rare diseases. First, the feature space ensemble method is more resistant against the selection bias of a single model, ensuring that a feature is not selected or excluded by coincidence. Second, the sample space ensemble is more data effective: When data are insufficient, an ensemble model is more stable and less likely to overfit. Besides, both ensemble techniques provide robust estimations compared with any single model and take advantage of different model structures (17). Third, only a few critical features are passed to the final model through feature selection to prevent overfitting (18). Taken together, these strategies alleviate the curse of dimensionality and the risk of overfitting, allowing effective models to be trained with limited data, as in rare diseases.

Our research has several limitations. It is important to emphasize that sEBV^+^T/NK-LPD is an exceptionally rare disease. Based on the rough overall annual incidence of lymphoma (6 × 10^–5^) and the observed proportion of sEBV^+^T/NK-LPD (1%), the estimated incidence of the disease can be approximated at 6 × 10^–7^, corresponding to fewer than 1 case per million people per year (19). Despite the inherent limitations in data availability, the inclusion of the prospective cohort (2020 to 2024) provided additional temporal validation and confirmed the robustness and generalizability of the COLLAPSED system.

Owing to its retrospective nature, the analysis relied solely on documented data, which reflect only the essential assessments and clinical understanding available at the time. Consequently, biomarkers such as soluble CD25 and other T cell activation markers, potentially influencing prognosis, could not be included. Although the COLLAPSED score has been validated using multiple independent datasets containing nationwide and worldwide patients, further validation, particularly with prospective international cohorts, remains important to enhance the applicability of the score. We strongly advocate international collaboration on prospective research to further evaluate the system in the care of patients with sEBV^+^T/NK-LPD globally. Nonetheless, our study represents an advancement by leveraging existing extensive data to generate valuable insights and set the foundation for future research.

In conclusion, we constructed the COLLAPSED system, which offers a practical and evidence-based approach to assessing the risk of patients with sEBV^+^T/NK-LPD. Using ML strategies, the system unbiasedly selects and incorporates 7 key factors to provide accurate, individualized risk assessments for patients. By categorizing patients into low- and high-risk groups, clinicians are better equipped to make informed decisions on treatment and monitoring, leading to timely interventions and paving the way for better outcomes in these patients.

## Methods

### Sex as a biological variable.

Female and male participants were enrolled in this study. The proposed system is applicable to patients of both sexes.

### Patients.

The study used data from the Lymphoproliferative Diseases/Lymphoma Database of the National Regional Pathology Consultation Center of China, covering the period from January 1, 2009, to December 31, 2019 (*n* = 18,571). The primary cohort of sEBV^+^T/NK-LPD was identified by screening of the database. All suspected cases were re-reviewed by 4 hematopathologists and diagnosed based on the WHO classification from 2017 and detailed descriptions from our previous studies, including the entities of chronic active EBV infection of T and NK cell type, systemic form (CAEBVD-T/NK-S), severe HV in HV-like LPD, and STLC (5, 6, 20, 21). These entities correspond to sCAEBVD, sHV-LPD, and STLC, respectively (2022 International Consensus Classification and WHO Classification, 5th edition) (1, 2). The endpoint was defined as the time from biopsy (time 0) to death due to the disease, while surviving patients were censored at the last date of contact. Patients were excluded by the following criteria: lacking follow-up data; concurrent with another tumor; or dead due to causes other than the disease (Supplemental Figure 3). Patients who underwent HSCT were included only for validation, because they usually achieved curable outcomes and changed their clinical course completely, making the estimation of the triage value of pre-treatment variables infeasible (22).

### Feature extraction.

We organized a multidisciplinary committee of hematopathologists, radiologists, pediatricians, and hematologists to discuss candidate features and include all feasible aspects in the selection process. Clinical features were collected from medical records. Pathological features were obtained via the reviewing process (antibodies used for immunostaining are summarized in Supplemental Table 6). Imaging features were gathered through images or radiological reports. If images were available, 3 specialists reviewed them. The distribution of ALN was assessed using 2 methodologies. First, we used the renowned Lugano classification, herein referred to as the “distribution of ALN” (23). Additionally, we devised an adaptation method, termed “distribution of ALN+.” In this corrected version, we define an “ALN group” as any collection with 1 or more ALN with the longest diameter greater than 1.5 cm, as outlined by the Lugano classification, or those showcasing a notable increase in small LNs. The latter marker serves as a potentially novel indicator for evaluating disease involvement based on empirical observations. Next, the distribution of ALN groups was stratified based on their anatomical positioning: either unilateral to the diaphragm, bilateral, or in a disseminated pattern. We did not incorporate “bulky disease” as an additional staging class in the distribution of ALN as in other primary nodal lymphomas; instead, we extracted it as an independent term for feature selection. The feature extraction process was blinded from the patient’s survival. To rigorously measure the model performance, the validation dataset was sealed until the model was finalized.

### Dataset generation.

The study included 4 independent datasets: (a) a primary retrospective multicenter cohort (*n* = 156, 2009–2019) for model development; (b) a prospective validation cohort (*n* = 35, 2020–2024) for temporal validation; (c) a transplant extension cohort used to evaluate model performance in patients who underwent HSCT; and (d) a literature validation cohort derived from published studies for external replication.

The primary retrospective cohort of patients with sEBV^+^T/NK-LPD (2009 to 2019) was split into a training dataset and a validation dataset using randomized stratified sampling, based on the year of diagnosis without replacement. The prospective cohort consisted of patients newly enrolled between January 1, 2020, and December 31, 2024, at West China Hospital. The inclusion and exclusion criteria were identical to those applied to the primary cohort. A total of 35 eligible patients were included in this independent validation cohort (Supplemental Figure 4).

The literature review was conducted using PubMed (2009 to 2020) and followed the same method as previously described (24). The workflow and included references are shown in Supplemental Figure 4. In total, 45 cases from 28 studies were included for analysis, which were from 11 countries: China (8 studies), Japan (8 studies), Korea (3 studies), the United States (2 studies), and Canada, Spain, Brazil, France, Oman, Iran, and Malaysia (1 study from each). The 45 cases from the literature review belonged to a variety of ethnic groups or communities (35 Asian, 7 Hispanic/Latino, 2 White, and 2 not reported) (Supplemental Figure 4, list of references for 28 studies included for validation). We checked author affiliations and key patient details (age, sex, clinical signs) to ensure no literature-mined cases were duplicated.

To determine whether the patterns of features selected by the COLLAPSED system in transplant patients also appear in some non-transplant patients (selection bias), and to assess the system’s discriminatory capacity on a mixed dataset, we included 8 patients from 8 studies who had undergone HSCT (Supplemental Figure 4, including references for the included publications). Along with our patients, those who underwent HSCT formed an extension dataset. To evaluate any selection bias, the pretreatment characteristics of these patients were juxtaposed with the characteristics of those who did not receive HSCT using hierarchical clustering analysis with Ward’s method. The distance was defined as 1 minus the Spearman’s correlation coefficient.

### Feature selection.

Before the feature selection was conducted, the missing data were filled in with the median of the corresponding feature in the training dataset. Then we applied feature selection to reduce the dimensionality of predictors and chose the optimum combination of factors. We adopted an integrated approach that combined the ensemble technique and SHAP value, representing the impact of features on model prediction (25). Practically, we performed the feature selection on a degraded ensemble of 3 base algorithms in the feature space. To analyze the link between survival and variables, we applied 3 base algorithms: Cox regression with an ELN, RSF, and SNN (26–28). Briefly, the ELN model estimates the parameters via solving:


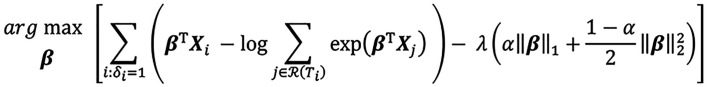


where *λ* controls the overall penalty magnitude; *α* ∈ [0,1] controls the trade-off between ℓ^1^ and ℓ^2^ regularization; and ℛ(*T_i_*) denotes the set of patients still at risk at time *T_i_*. The RSF comprises an ensemble of *B* survival trees:


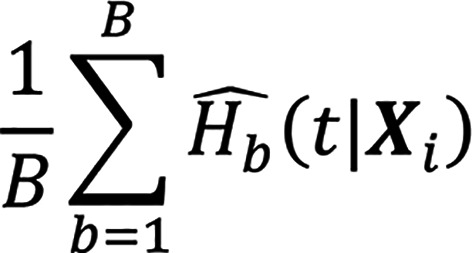


where 
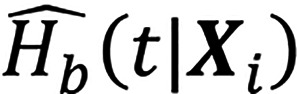
 is the cumulative hazard function estimated via the Nelson-Aalen estimator. For the SNN model, a multilayer perceptron with 1 hidden layer (100 hidden neurons) was used, and regularization weights and random initializations were varied in 100 trials. The loss function was defined as:


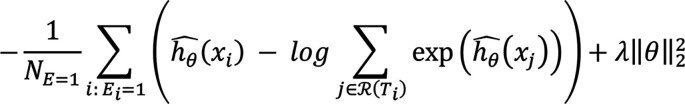


where 
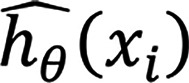
 is the network’s output that estimates the log-risk function; NE=1 is the number of patients with an observable event; ℛ(*T_i_*) denotes the risk set; and *λ* is the regularization hyperparameter controlling the strength of the ℓ^2^ penalty.

For each algorithm *a* ∈ A = {*ELN*,*RSF*,*SNN*}, we define a set of hyperparameter configurations 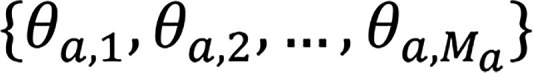. Each trial (*a*,*m*) is a unique combination of hyperparameters and is trained with 1,000 different parameter combinations each, and the 5-fold cross-validation model performance was evaluated based on the index of prediction accuracy on the training dataset (29). For ELN and RSF, the top 100 models out of the 1,000 were preserved. In the preserved trials, the SHAP importance of each feature was calculated, and only the 10 most critical features were selected. Let SHAP_a,m_(*j*) represent the mean absolute SHAP value of feature *j* in trial (*a*, *m*); the set of indices of the top 10 features for algorithm *a* in trial *m* is:


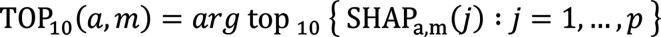


The selection frequency was used as the primary reference statistic. For a given algorithm *a*, the selection frequency of feature *j* is:


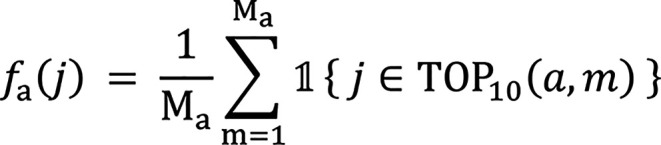


where 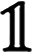 is the indicator function. A feature was selected if it was one of the top 10 features in more than 60% (τ = 0.6) of any base algorithm and was selected by at least 2 of the 3 base algorithms. The final set of selected features is:


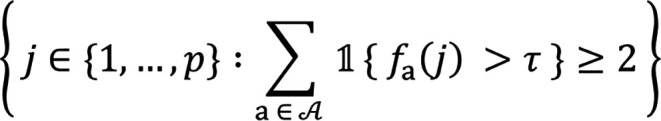


### COLLAPSED model generation and evaluation.

With the selected 7 features, we trained ELN, RSF, and SNN models and averaged the best model within each algorithm to generate the ensemble model, which is:


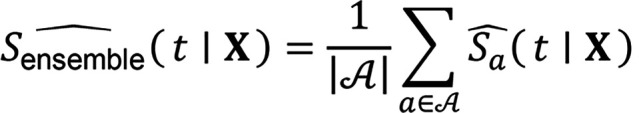


where 
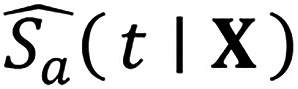
is the survival estimate from the model *a*.

To evaluate the internal performance of the models, a 5-fold cross-validation was conducted 3 times for ELN and RSF, and a pre-divided test set from the training dataset was used for SNN. The internal performance was determined by the index of prediction accuracy. To evaluate the model externally, we calculated the censoring-adjusted C statistic at 6 months and 12 months as the overall discrimination measurement (30). The areas under the time-dependent receiver operating characteristic (ROC) curves (AUCs) were computed to reflect the dynamic changes in discrimination ability (31). To evaluate the calibration ability of the model, we depicted graphical calibration curves and calculated the integrated calibration index, which is the weighted difference between smoothed observed proportions and predicted probabilities, and E90, which denotes the 90th percentile of the absolute difference between observed and predicted probabilities (32). All the measurements were calculated on the validation dataset, and the corresponding 95% CIs were estimated through bootstrapping resampling methods (with 10,000 repetitions).

### COLLAPSED score specification and evaluation.

To assess the model’s utilization of inputs and generate a streamlined risk score for clinical purposes, we calculated the SHAP value, which explains the individual predictions on the training dataset. For each feature, we allocated samples with similar SHAP values into bins. If a bin had limited patient samples, we merged it with its adjacent bins. Additionally, the clinical reference value was used to determine certain boundaries, which helped distinguish the normal variation from the diseases. Finally, to assign a score to each bin, we divided the mean SHAP value within it by 0.25 and rounded the result to the nearest integer. Then, the risk group was generated by dichotomizing of the total score: low risk (total score ≤ 7) and high risk (total score > 7). The cutoff for the risk score was determined by selection of the point on the ROC curve closest to the ideal coordinate (0,1) for predicting the median survival in the construction cohort. The Kaplan-Meier estimator was used to illustrate the probability of death due to the disease. The log-rank test was performed to compare death probabilities between groups.

### Subgroup analysis.

Given the limited number of patients in each subgroup in the validation dataset, we calculated the C index of the model for the following subgroups in the combined dataset (training and validation datasets) with bootstrapped corrections. The subgroups were specified based on patients’ age (≤14 and >14), year of diagnosis (2009–2013, 2014–2017, and 2018–2019), and the primary treatment received: CHOP/CHOP-like (cyclophosphamide, vincristine, adriamycin, and prednisone), ED (etoposide, dexamethasone), and l-asparaginase–based (L-based) protocols, chemotherapy followed by programmed cell death protein 1 blockade (chemotherapy + PD-1 blockade), glucocorticoid, and antiviral therapy. The subgroups of treatment and year of diagnosis were predetermined, while the subgroup of age was determined post hoc as age was not included in the model.

### Statistics.

All the analyses were formed in R (v4.1.0) or Python (v3.9.5). The data visualization was done by ggplot2 (v3.3.5). Differences in cumulative probability of death between groups were assessed using the log-rank test. For baseline comparisons, the Kruskal-Wallis rank-sum test was used for continuous variables, and Pearson’s χ^2^ test was used for categorical variables. The association between the model output and score was assessed using Spearman’s rank correlation test. A 2-sided *P* less than 0.05 was considered statistically significant unless otherwise specified.

### Study approval.

This study was approved by the Ethics Committee on Biomedical Research at West China Hospital of Sichuan University (2020-1016) and was exempt from informed consent. This study was registered at the Chinese Clinical Trial Registry (https://www.chictr.org.cn) as ChiCTR2000039655.

### Data availability.

All relevant data are available in the article, supplement, or Supporting Data Values file or upon reasonable request.

## Funding support

- National Natural Science Foundation of China (81900195 to ZC).
- 1·3·5 project for disciplines of excellence — Clinical Research Incubation Project, West China Hospital, Sichuan University (2019HXFH001 to W Liu and 2021HXFH027 to SZ).
- Post-Doctor Research Project, West China Hospital, Sichuan University (2019HXBH067 to ZC).

## Author contributions

PG and ZC designed the study. ZC, PG, XG, JH, TD, MW, XL, and LZ contributed to gathering or providing the clinical data. ZC, WZ, SZ, and W Liu reviewed the pathological slides. PG, W Li, and RL reviewed the imaging materials. PG and HD performed the data analysis and built the model; SW supervised the data analysis. PG and ZC wrote the paper. YT, FH, LP, JG, SW, RL, WZ, SZ, and W Liu revised the paper. SZ and W Liu supervised the study. The order of the co–first authors (PG and ZC) was decided based on their equal contribution and mutual agreement. All authors approved the manuscript.

## Supplementary Material

Supplemental data

Supporting data values

## Version 1 02/23/2026

Electronic publication
