# Supplementary material for: A machine learning–based triage system for systemic EBV-positive T/NK cell lymphoproliferative diseases of childhood
Source: JCI Insight. 2026 Feb 23;11(4):e180837. doi: 10.1172/jci.insight.180837 (PMC12955998; doi:10.1172/jci.insight.180837)
Supplement: Supplemental data [file jciinsight-11-180837-s261.pdf]

A machine learning-based triage system for systemic EBV-positive T/NK cell lymphoproliferative diseases of childhood

Pujun Guan, Zihang Chen, Hanze Dong, Xia Guo, Juan Huang, Tian Dong, Mi Wang, Xiaoxi Lu, Fei Huang, Wenbin Li, Yuan Tang, Li Zhang, Ling Pan, Ju Gao, Shikun Wang, Rongbo Liu, Wenyan Zhang, Sha Zhao, Weiping Liu

Supplementary Tables and Figures

Table s1: Baseline characteristics of patients with sEBV+T/NK-LPDs in the primary retrospective cohort

Table s2: Baseline characteristics of patients with sEBV+T/NK-LPDs in the prospective validation cohort

Table s3: Checklist for calculating the COLLAPSED score

Table s4: Quantitative evaluation of model calibration

Table s5: Bootstrapped C-index values across subgroups for the ensemble model

Table s6: Antibodies used for immunostaining

Figure s1: Radiological and pathological features of abnormal lymph nodes+ (ALN+)

Figure s2: Calibration and interpretability analyses of the COLLAPSED system

Figure s3: Triage flowchart for patients with sEBV^{+}T/NK-LPD based on the COLLAPSED system

Figure s4: Overview of cohorts included in this study

Table s1: Baseline characteristics of patients with sEBV+T/NK-LPDs in the primary retrospective cohort

	Retrospective cohort, n (%)			
**Characteristics**	**All (N=156)**	**Training (N=94)**	**Validation (N=62)**	**P value^**
Clinical Findings				
***Age at Diagnosis, median(range), y**	24 (1-72)	23 (1-72)	26 (2-51)	0.753
**Sex (M/F)**	101/55	61/33	40/22	0.962
**Disease Group**				0.398
sCAEBVD	128 (82.1)	79 (84.0)	49 (79.0)	
sHV-LPD	8 (5.1)	3 (3.2)	5 (8.1)	
STLC	20 (12.8)	12 (12.8)	8 (12.9)	
***Course, median(range), mth**	4.0 (0.3-156.0)	4.8 (0.3-156.0)	3.0 (0.3-36.0)	0.015
***Fever**				0.937
NA	0	0	0	
Present	138 (88.5)	83 (88.3)	55 (88.7)	
***Night Sweat**				0.404
NA	2	1	1	
Present	14 (9.1)	7 (7.5)	7 (11.5)	
***Lose Weight**				0.072
NA	2	1	1	
Present	23 (14.9)	10 (10.8)	13 (21.3)	
***B-symptoms**				0.564
NA	0	0	0	
Present	141 (90.4)	86 (91.5)	55 (88.7)	
***PS**				0.051
NA	0	0	0	
0	44 (28.2)	30 (32.0)	14 (22.6)	
1	59 (37.8)	32 (34.0)	27 (43.5)	
2	15 (9.6)	13 (13.8)	2 (3.2)	
3	29 (18.6)	13 (13.8)	16 (25.8)	
4	9 (5.8)	6 (6.4)	3 (4.8)	
***Concomitant Diseases**				0.803
NA	0	0	0	
Present	14 (9.0)	8 (8.5)	6 (9.7)	
***HLH**				0.208
NA	3	1	2	
Present	77 (50.3)	43 (46.2)	34 (56.7)	
Imaging Findings				
***Splenomegaly**				0.655
NA	0	0	0	
Present	126 (80.8)	77 (81.9)	49 (79.0)	
***Hepatomegaly**				0.950
NA	0	0	0	

Present	75 (48.1)	45 (47.9)	30 (48.4)	
***Extranodal Organ Involvement**				0.981
NA	3	1	2	
Present	120 (78.4)	73 (78.5)	47 (78.3)	
***Distribution of ALN**				0.731
**(Defined by Lugano classification)**				
NA	2	1	1	
None	36 (23.4)	25 (26.9)	11 (18.0)	
Regional	37 (24.0)	20 (21.5)	17 (27.9)	
One side of diaphragm	46 (29.9)	28 (30.1)	18 (29.5)	
Both sides of diaphragm	26 (16.9)	15 (16.1)	11 (18.0)	
Disseminated	9 (5.8)	5 (5.4)	4 (6.6)	
***Distribution of ALN+**				0.160
NA	2	1	1	
One side of diaphragm	31 (20.1)	22 (23.7)	9 (14.8)	
Both sides of diaphragm	56 (36.4)	36 (38.7)	20 (32.8)	
Disseminated	67 (43.5)	36 (38.7)	32 (52.5)	
***Bulk LN**				0.285
NA	2	1	1	
None	133 (86.4)	80 (86.0)	53 (86.9)	
3cm≤φ<6cm	14 (9.1)	7 (7.5)	7 (11.5)	
φ≥6cm	7 (4.5)	6 (6.5)	1 (1.6)	
***Effusions**				0.232
NA	2	1	1	
None	96 (62.3)	62 (66.7)	34 (55.7)	
1 site	26 (16.9)	12 (12.9)	14 (23.0)	
≥2 sites	32 (20.8)	19 (20.4)	13 (21.3)	
Laboratory Findings				
***WBC**				0.362
NA	2	1	1	
median (range), 10^9/L	3.69 (0.05-27.04)	3.80 (0.05-27.04)	3.37 (0.21-16.94)	
***RBC**				0.773
NA	2	1	1	
median (range), 10^{12}/L	3.74 (1.41-7.30)	3.77 (1.41-6.40)	3.73 (1.93-7.30)	
***Hb**				0.756
NA	2	1	1	
median (range), g/L	102 (44-162)	102 (46-162)	102 (44-159)	
***PLT**				0.643
NA	2	1	1	
median (range), 10^9/L	130 (4-806)	132 (8-494)	114 (4-806)	
***NEUT**				0.675
NA	2	1	1	
median (range), 10^9/L	2.19 (0.02-23.88)	2.22 (0.02-23.88)	2.11 (0.04-13.50)	
***LYMPH**				0.231

NA	2	1	1	
median (range), 10^9/L	0.93 (0.01-6.84)	0.97 (0.01-6.84)	0.86 (0.04-4.95)	
***NLR**				0.728
NA	2	1	1	
median (range)	2.29 (0.17 -54.17)	2.18 (0.10-42.50)	2.40 (0.10-54.17)	
***EO**				0.913
NA	16	11	5	
median (range), 10^9/L	0.01 (0.00-1.88)	0.01 (0.00-1.88)	0.01 (0.00-1.61)	
***AST**				0.792
NA	6	3	3	
median (range), IU/L	56 (8-2142)	56 (8-2142)	55 (13-614)	
***ALT**				0.133
NA	6	3	3	
median (range), IU/L	43 (9-668)	38 (9-611)	53 (7-668)	
***AST/ALT**				0.196
NA	6	3	3	
median (range)	1.24 (0.27-4.84)	1.26 (0.29-4.84)	1.12 (0.27-3.07)	
***Total Bilirubin**				0.380
NA	6	3	3	
median (range), μmol/L	11.7 (3.8-240.9)	11.2 (3.8-136.1)	12.7 (3.8-240.9)	
***Direct Bilirubin**				0.508
NA	6	3	3	
median (range), μmol/L	5.6 (1.2-209.2)	5.2 (1.3-119.1)	5.9 (1.2-209.2)	
***ALP**				0.738
NA	6	3	3	
median (range), IU/L	124 (23-1479)	125 (23-1479)	121 (36-1214)	
***LDH**				0.723
NA	6	3	3	
median (range), IU/L	428 (113-7800)	428 (113-7800)	427 (138-2290)	
***ALB**				0.975
NA	6	3	3	
median (range), g/L	34.2 (16.7-48.3)	34.3 (20.0-48.3)	33.4 (16.7-46.3)	
***GLB**				0.350
NA	6	3	3	
median (range), g/L	25.8 (10.0-62.5)	26.1 (12.8-47.9)	24.3 (10.0-62.5)	
***TG**				0.761
NA	8	5	3	
median (range), mmol/L	1.80 (0.59-20.73)	1.83 (0.73-6.83)	1.77 (0.59-20.73)	
***Fibrinogen**				0.701
NA	17	9	8	
median (range), g/L	2.02 (0.48-6.58)	2.12 (0.48-6.58)	1.91 (0.50-5.81)	
***Plasma EBV-DNA (log10)**				0.542
NA	27	17	10	
median (range),	4.16 (1.70-7.79)	4.23 (1.70-7.79)	4.00 (2.32-7.00)	

Pathological Findings				
***Local structure**				0.095
Immeasurable	35 (22.4)	18 (19.1)	17 (27.4)	
Preserved	29 (18.6)	22 (23.4)	7 (11.3)	
Destroyed	92 (60.0)	54 (57.4)	38 (61.3)	
***Necrosis Site**				0.983
Subcapsular Necrosis	15 (9.6)	9 (9.6)	6 (9.7)	
Paracortical Necrosis	26 (16.7)	15 (16.0)	11 (17.7)	
***Necrosis pattern**				0.067
Focal Necrosis	20 (12.8)	13 (13.8)	7 (11.3)	
Patchy Necrosis	18 (11.5)	9 (9.6)	9 (14.5)	
Irregular Necrosis	6 (6.4)	2 (3.2)	8 (5.1)	
***Necrosis Rate, median (range), %**	0 (0-60)	0 (0-60)	0 (0-60)	0.986
***Hyperplasia Pattern**				0.252
Interfollicular Hyperplasia	80 (51.3)	48 (51.1)	32 (51.6)	
Submucosal Hyperplasia	13 (8.3)	7 (7.4)	6 (9.7)	
Diffuse Hyperplasia	32 (20.5)	24 (25.5)	8 (12.9)	
***Vascular infiltration**	19 (12.2)	11 (11.7)	8 (12.9)	0.822
***Vascular Hyperplasia**	33 (21.2)	21 (22.3)	12 (19.4)	0.655
***Cell Size**				0.864
Small	12 (19.4)	17 (18.1)	14 (22.6)	
Small-Medium	40 (25.6)	24 (25.5)	16 (25.8)	
Medium	48 (30.8)	28 (29.8)	20 (32.3)	
Medium-Large	28 (17.9)	19 (20.2)	9 (14.5)	
Large	9 (5.8)	6 (6.4)	3 (4.8)	
***Cell Morphic**				0.868
RS-like	11 (7.1)	7 (7.4)	4 (6.5)	
Polymorphic	132 (84.6)	80 (85.1)	52 (83.9)	
Monomorphic	13 (8.3)	7 (7.4)	6 (9.7)	
***Mitosis, median (range), /HP**	1 (0-12)	1 (0-7)	0 (0-12)	
***Apoptosis**	53 (34.0)	31 (33.0)	22 (35.5)	0.746
***Histocyte Hyperplasia**	41 (26.3)	26 (27.7)	15 (24.2)	0.630
***Erythrophagocytosis**	24 (15.4)	15 (16.0)	9 (14.5)	0.807
***Cell Origin**				0.905
T	95 (60.9)	58 (61.7)	37 (59.7)	
T/NK	31 (19.9)	19 (20.2)	12 (19.4)	
NK	30 (19.2)	17 (18.1)	13 (21.0)	
***EBER, median (range), %**	40 (1-80)	40 (1-80)	40 (1-80)	0.881
**CD3**				1.000
NA	0	0	0	
Positive	156 (100)	94 (100)	62 (100)	
**CD20**				1.000
NA	0	0	0	
Positive	0 (0)	0 (0)	0 (0)	

**TIA-1**				1.000
NA	3	1	2	
Positive	153 (100)	93 (100)	60 (100)	
***Granzyme B**				0.945
NA	13	8	5	
Positive	115 (80.4)	69 (80.2)	46 (80.7)	
***CD5**				0.512
NA	29	19	10	
Positive	80 (63.0)	49 (65.3)	31 (59.6)	
***CD4**				0.403
NA	23	16	7	
Positive	79 (59.4)	44 (56.4)	35 (63.6)	
***CD8**				0.964
NA	23	16	7	
Positive	97 (72.9)	57 (73.1)	40 (72.7)	
***CD30**				0.850
NA	35	20	15	
median (range), %	5 (0-100)	5 (0-100)	10 (0-100)	
***Ki-67**				0.778
NA	25	14	11	
median (range), %	50 (5-90)	50 (6-90)	50 (5-90)	
***TR* Rearrangement**				0.943
NA	23	13	10	
Monoclonal	43 (32.3)	26 (32.1)	17 (32.7)	
**Treatment**				
CHOP/CHOP-like regimen	22 (14.1)	14 (14.8)	8 (12.9)	0.727
ED regimen	26 (16.7)	19 (20.2)	7 (11.3)	0.143
L-based regimen	15 (9.6)	7 (7.4)	8 (12.9)	0.258
Other chemotherapy	10 (6.4)	5 (5.3)	5 (8.1)	0.493
Chemotherapy+PD-1 blockade	16 (10.3)	6 (6.4)	10 (16.1)	0.050
Glucocorticoid	41 (26.3)	25 (26.6)	16 (25.8)	0.202
Antiviral therapy	16 (10.3)	11 (11.7)	5 (8.1)	0.637
Supportive care	10 (6.4)	7 (7.4)	3 (4.8)	0.464

* The candidate features

^ Kruskal-Wallis rank-sum test was used for numeric variables, and Pearson's Chi-squared test was used for categorical variables.

y: year; M: male; F: female; sCAEBVD: systemic chronic active EBV disease; STLC: systemic EBV+T-cell lymphoma of childhood; sHV-LPD: systemic hydroa vacciniforme lymphoproliferative disorder; mth: month, NA; not available; PS: performance status; HLH: hemophagocytic lymphohistiocytosis; ALN: abnormal lymph node; WBC: white blood cell; RBC: red blood cell; Hb: hemoglobin; PLT: platelet; NEUT: neutrophils; LYMPH: lymphocyte; EO: eosinophils; AST: aspartate aminotransferase; ALT: alanine aminotransferase; ALP: alkaline phosphatase; LDH: lactate dehydrogenase; ALB: albumin; GLB: globulin; TG: triglyceride; TR: T-cell receptor

Table s2: Baseline characteristics of patients with sEBV+T/NK-LPDs in the prospective validation cohort

Characteristics	Retrospective cohort N=156; n(%)	Prospective cohort N=35; n(%)	P value^
Clinical Findings			
**Age at Diagnosis, median(range), y**	24 (1-72)	27 (13-57)	0.233
**Sex (M/F)**	101/55	17/18	0.075
**Disease Group**			0.769
sCAEBVD	128 (82.1)	29 (82.9)	
sHV-LPD	8 (5.1)	3 (8.6)	
STLC	20 (12.8)	5 (14.3)	
**PS**			0.001
NA	0	0	
0	44 (28.2)	2 (5.7)	
1	59 (37.8)	11 (31.4)	
2	15 (9.6)	9 (25.7)	
3	29 (18.6)	13 (37.1)	
4	9 (5.8)	0 (0)	
Imaging Findings			
**Distribution of ALN+**			0.002
NA	2	0	
One side of diaphragm	31 (20.1)	2 (5.7)	
Both sides of diaphragm	56 (36.4)	24(68.6)	
Disseminated	67 (43.5)	9 (25.7)	
**Effusions**			0.031
NA	2	0	
None	96 (62.3)	12 (34.3)	
1 site	26 (16.9)	13 (37.1)	
≥2 sites	32 (20.8)	10 (28.696)	
Laboratory Findings			
**LYMPH**			0.239
NA	2	0	
median (range), 109/L	0.93 (0.01-6.84)	0.78 (0.03-8.91)	
**AST**			0.911
NA	6	0	
median (range), IU/L	56 (8-2142)	48 (9-775)	
**LDH**			0.614
NA	6	0	
median (range), IU/L	428 (113-7800)	425 (122-4560)	
**Cell Origin**			0.289
T	95 (60.9)	17 (48.6)	
T/NK	31 (19.9)	11 (31.4)	
NK	30 (19.2)	7 (20.0)	

^ Kruskal-Wallis rank-sum test was used for numeric variables, and Pearson's Chi-squared test was used

for categorical variables.

y: year; M: male; F: female; sCAEBVD: systemic chronic active EBV disease; STLC: systemic EBV+T-cell lymphoma of childhood; sHV-LPD: systemic hydroa vacciniforme lymphoproliferative disorder; mth: month, NA; not available; PS: performance status; ALN: abnormal lymph node; LYMPH: lymphocyte; AST: aspartate aminotransferase; LDH: lactate dehydrogenase

Table s3: Checklist for calculating the COLLAPSED score

Name	Age	Sex	ID	Diagnosis Date

COLLAPSED SCORE CHECKLIST

Terms	Test		Score	Mark
**Cell Origin**	Histopathological and/or flow cytometry analysis of biopsied tissue	NK	0	
		T/NK	1	
		T	2	
**Lymphocyte Count ($\times 10^9$/L)**	Blood routine test (The most recent test before biopsy.)	>3	0	
		3≤n<1	1	
		1≤n<0.5	2	
		≤0.5	3	
**LDH (IU/L)**	Blood biochemistry test (The most recent test before biopsy.)	<440	0	
		≥440	1	
**AST (IU/L)**	Blood biochemistry test (The most recent test before biopsy.)	<55	0	
		≥55	1	
**ECOG Performance Status**	Physical test	0	0	
		1	1	
		2 - 4	2	
**Effusions**	Whole-body CT/MRI is preferred. (Ultrasonography and physical test could be used as a complement.)	Absent	0	
		Present	1	
**Distribution of Abnormal Lymph Node (plus)**	Whole-body contrast-enhanced CT/MRI is preferred. (Ultrasonography and physical test could be used as a complement.)	One side	0	
		Both sides	2	
		Disseminated	4	

**Total Score**		**Low-risk Group** (Total score≤7)		**High-risk Group** (Total score>7)	

**Doctor Signature**		**Date**	

Notice:

The system is for scientific usages only. Practitioners and researchers must always rely on their own experience and knowledge in evaluating and using the system described herein. To the fullest extent of the law, no responsibility is assumed by the authors or contributors for any injury and/or damage to persons or property from any use of the system herein.

Term definition

Cell Origin

NK origin: (Fulfill one of the following criteria)

1. Pleomorphic or abnormal antigen-expressed EBV-infected NK-cell in biopsied tissue without any T-cell abnormality (Evaluated by histopathological or flow cytometry analysis)
2. Obvious EBV-infected NK-cell proliferation in histopathological analysis without any T-cell abnormality.

T/NK origin: (Fulfill one of the following criteria)

1. Proliferation of both EBV-infected T-cell and NK-cell in biopsied tissue without any abnormality in cytology or antigen expression (Evaluated by histopathological or flow cytometry analysis)
2. Abnormality in both EBV-infected T-cell and NK-cell (Evaluated by histopathological or flow cytometry analysis)
3. Proliferation of EBV-infected cells with atypical immunomarkers that cannot determine the origin from T-cell or NK-cell. (Evaluated by histopathological or flow cytometry analysis)

T origin: (Fulfill one of the following criteria)

1. Monoclonal TR rearrangement
2. Undetected NK-cell in histological analysis or detected normal NK cell with normal proportion in flow cytometry analysis

ECOG PS*

Score 0: Fully active, able to carry on all pre-disease performance without restriction

Score 1: Restricted in physically strenuous activity but ambulatory and able to carry out work of a light or sedentary nature.

Score 2: Ambulatory and capable of all selfcare but unable to carry out any work activities. Up and about more than 50% of waking hours

Score 3: Capable of only limited selfcare, confined to bed or chair more than 50%of waking hours

Score 4: Completely disabled. Cannot carry on any selfcare. Totally confined to bed or chair.

** Lansky Scale is applied to pediatric patients who could not evaluated by ECOG PS*

Lansky scale 90-100 equal to ECOG PS 0; Lansky scale 70-80 equal to ECOG PS 1; Lansky scale 50-60 equal to ECOG PS 2;

Lansky scale 30-40 equal to ECOG PS 3; Lansky scale 10-20 equal to ECOG PS 4

Distribution of abnormal lymph node (LN) +

That a LN group contains one or more abnormal LN, or significant LN increase is considered as abnormal: (Fulfill one of the following criteria)

1. LN with the longest transverse diameter larger than 1.5 cm
2. Other evidence that confirms or highly suspects the LN involvement, including pathological, PET/CT, multi-modality MRI, ultrasonography, etc.
3. LN increases asymmetrically on one side relative to the other side, which cannot be explained by other causes
4. More than 4 LNs are found in a LN group, which cannot be explained by other causes
5. A LN is found in a region that usually shows no LNs, which cannot be explained by other causes
6. LN increases comparing with previous scan, which cannot be explained by other causes

Distribution of abnormal LN is defined as follows:

One side: Abnormal LN group on one side of diaphragm

Both sides: Abnormal LN group on both sides of diaphragm

Disseminated: Noncontiguous multiple extranodal involvement and/or abnormal lymph node groups in nearly every region in the body

Effusions

Minimal or more effusions are found in any regions by thoracic or abdominal imaging, which cannot be explained by other causes unrelated to sEBV+T/NK-LPD.

Table s4 Quantitative evaluation of model calibration

Model	12 months		8 months		4 months	
	ICI (95% CI)	E90 (95% CI)	ICI (95% CI)	E90 (95% CI)	ICI (95% CI)	E90 (95% CI)
**Ensemble**	0.097 (0.086-0.108)	0.161 (0.157-0.163)	0.065 (0.058-0.073)	0.115 (0.11-0.116)	0.054 (0.048-0.059)	0.085 (0.082-0.087)
**ELN**	0.068 (0.103-0.111)	0.112 (0.131-0.138)	0.051 (0.063-0.073)	0.081 (0.103-0.116)	0.07 (0.046-0.056)	0.098 (0.078-0.082)
**RSF**	0.07 (0.065-0.075)	0.098 (0.096-0.098)	0.042 (0.037-0.047)	0.079 (0.076-0.08)	0.046 (0.041-0.051)	0.077 (0.075-0.078)
**SNN**	0.092 (0.087-0.097)	0.135 (0.118-0.148)	0.052 (0.044-0.06)	0.128 (0.097-0.143)	0.088 (0.078-0.097)	0.143 (0.128-0.169)

Integrated calibration index (ICI): weighted difference between smoothed observed proportions and predicted probabilities. E90: the 90th percentile of the absolute difference between observed and predicted probabilities. ELN: Cox regression with elastic-net penalty. RSF: random survival forest. SNN: survival neural network.

Table s5 Bootstrapped C-index values across subgroups for the ensemble model

	N	Events	6 mths C-index (95% CI)	12 mths C-index (95% CI)
**Age**				
≤14	33	23	0.852 (0.725-0.959)	0.872 (0.763-0.957)
>14	123	93	0.832 (0.79-0.882)	0.821 (0.768-0.868)
**Year of diagnosis**				
2009-2013	52	47	0.899 (0.830-0.956)	0.904 (0.845-0.952)
2014-2017	65	49	0.771 (0.684-0.850)	0.782 (0.702-0.853)
2018-2019	39	20	0.897 (0.823-0.957)	0.825 (0.713-0.912)
**Treatment**				
Chemotherapy				
CHOP/CHOP-like	22	20	0.791 (0.600-0.936)	0.803 (0.654-0.924)
ED	26	21	0.830 (0.682-0.944)	0.790 (0.635-0.912)
L-based	15	12	0.861 (0.605-1.000)	0.807 (0.583-0.975)
Chemotherapy+PD-1 blockade	16	6	0.926 (0.688-1.000)	0.794 (0.534-0.978)
Glucocorticoid	56	36	0.856 (0.768-0.930)	0.855 (0.780-0.920)
Antiviral therapy	16	12	0.918 (0.785-1.000)	0.938 (0.809-1.000)

Table s6: Antibodies used for immunostaining

Antibody	Clonal	Manufacturer	Dilution
**CD20**	L26	DAKO	1:100
**CD3**	PS1	DAKO	1:100
**CD4**	1F6	Novocastra	1:50
**CD5**	4C7	Neomarkers	1:100
**CD8**	C8	DAKO	1:100
**CD30**	Ber-H2	Neomarkers	1:50
**CD56**	123C3	Zymed	1:100
**Granzyme B**	GZB01	Neomarkers	1:100
**TIA-1**	TIA-1	Zymed	1:50
**Ki-67**	MIB1	Neomarkers	1:150

Figure s1: Radiological and pathological features of abnormal lymph nodes+ (ALN+)

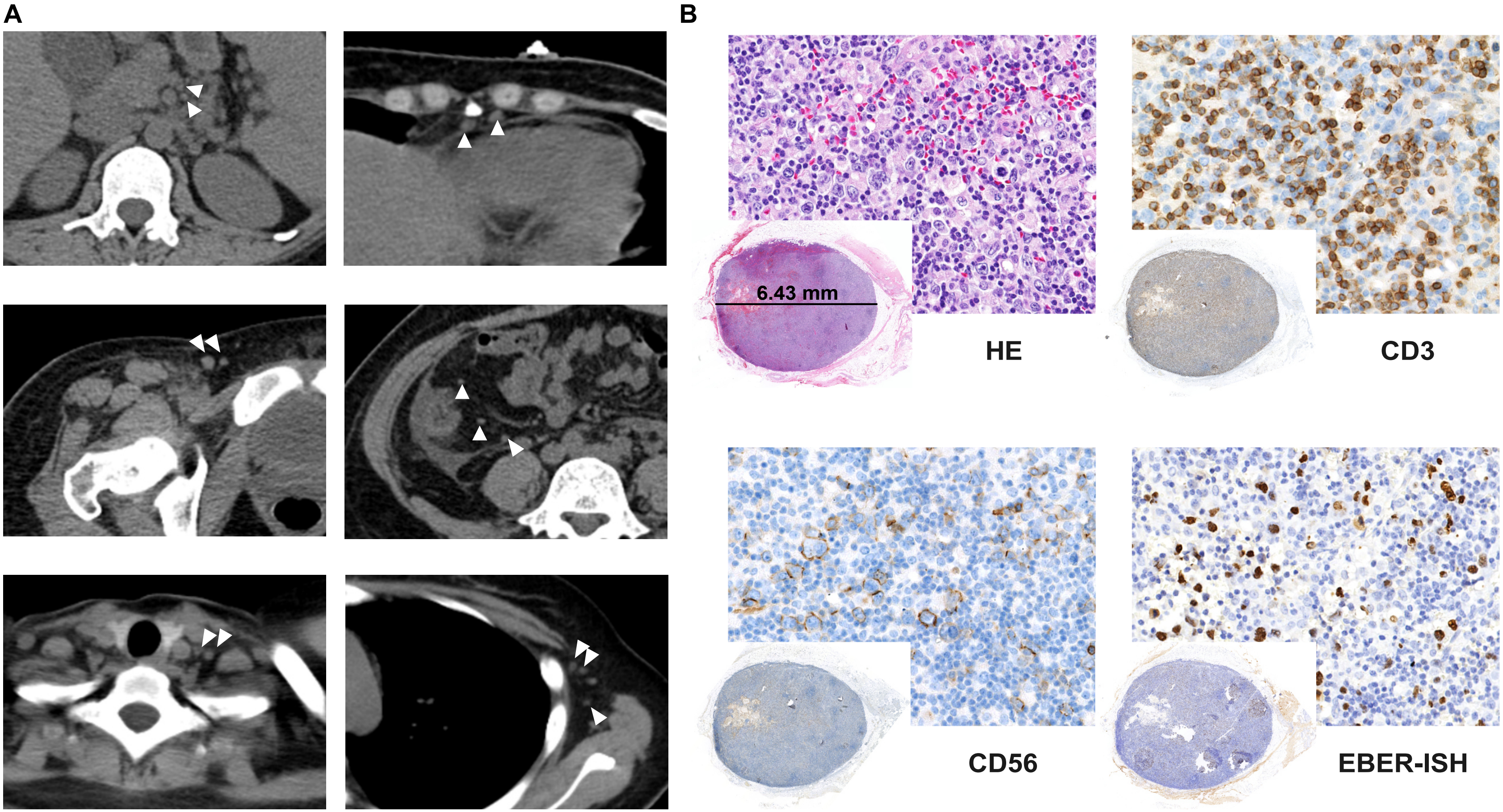

A. A rise in small lymph nodes across distinct anatomical regions. (In sequence: retroperitoneal, cardiophrenic angle, inguinal, mesenteric, supraclavicular, and axillary lymph nodes.)

B. Biopsy of a small lymph node demonstrated the infiltration of EBV-infected NK-cells, which were positive for CD3, CD56 and EBER1/2-ISH.

Figure s2: Calibration and interpretability analyses of the COLLAPSED system

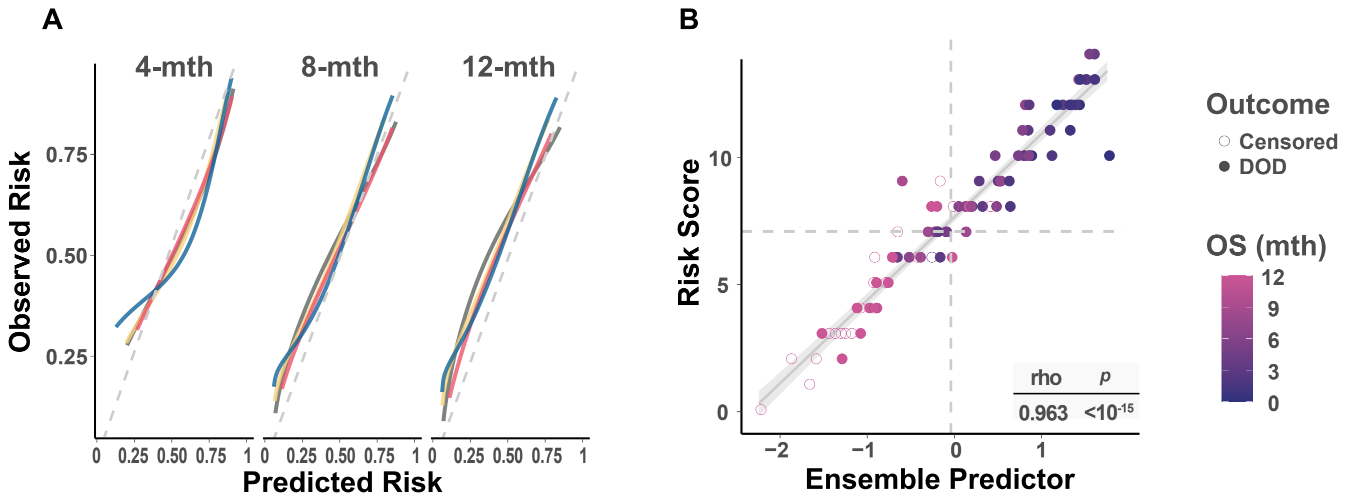

A. Calibration plots for the four models, comparing predicted risks with observed outcomes at 4, 8, and 12 months. The dashed line represents the ideal calibration curves.

B. Relationship between predictors from the ensemble model and assigned risk scores in the training data.

Figure s3: Triage flowchart for patients with sEBV+T/NK-LPD-C based on the COLLAPSED system

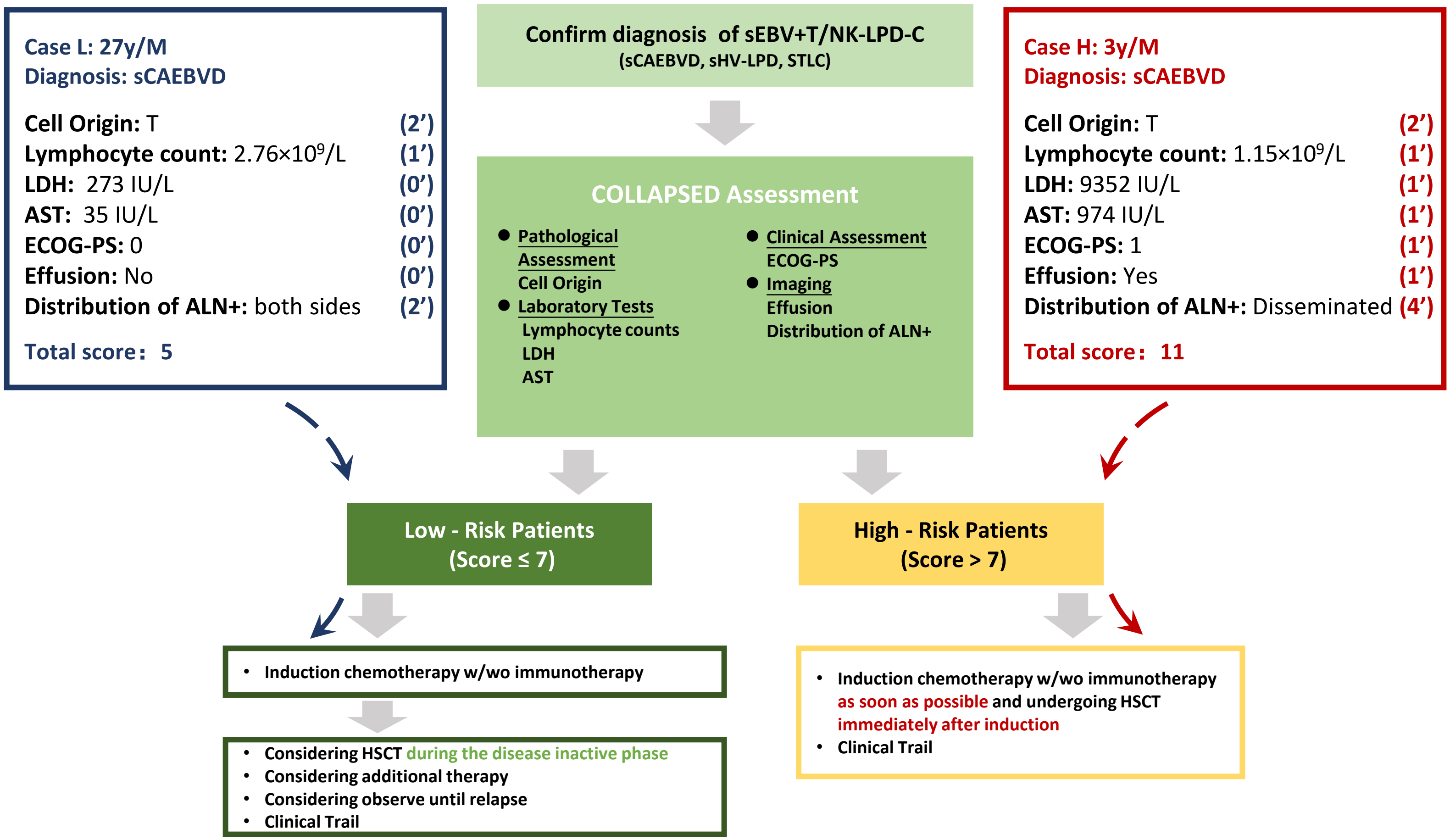

Figure s4: Overview of cohorts included in this study

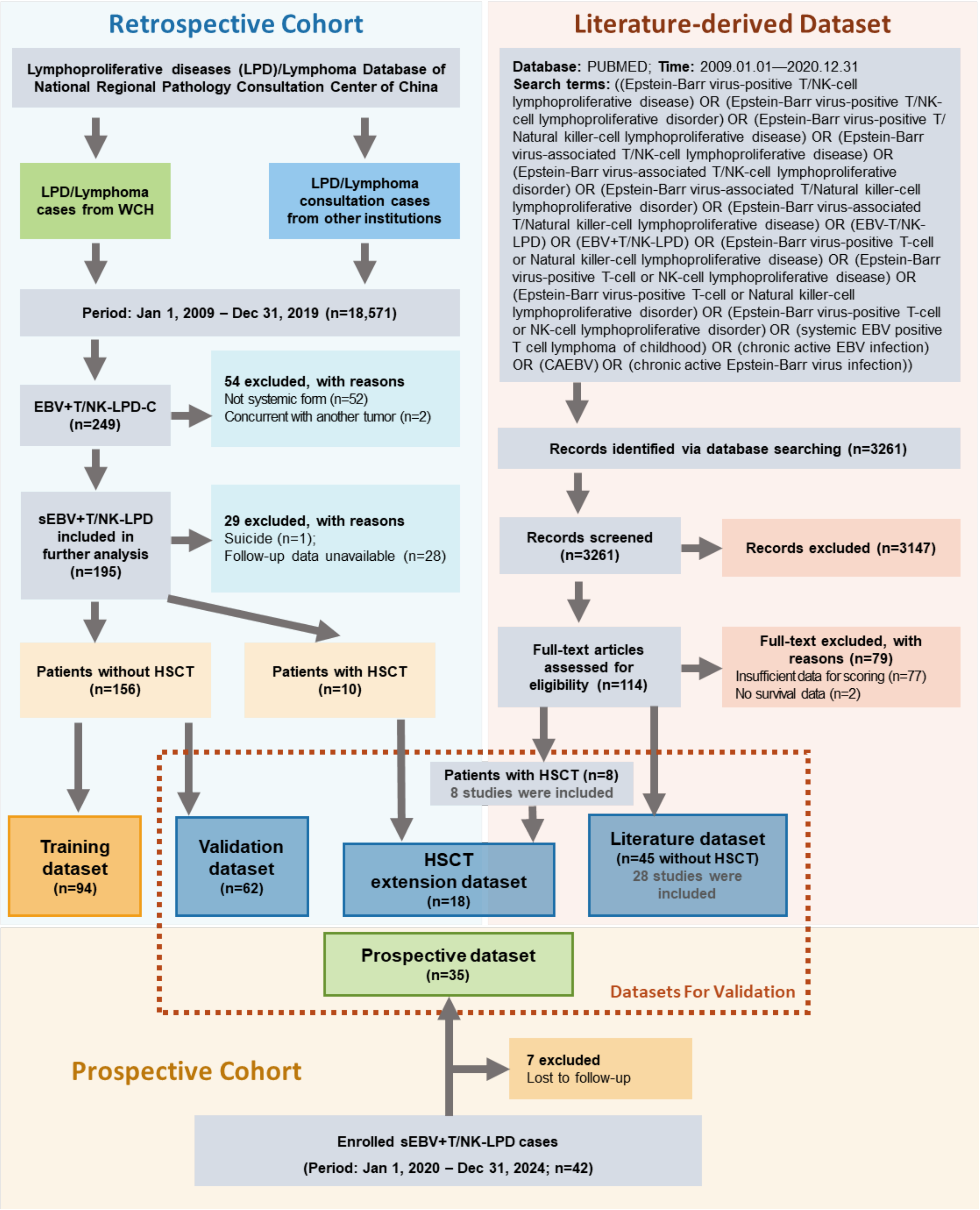

List of 28 studies included for external validation：

1. Hasegawa D, Kaji M, Takeda H, Kawasaki K, Takahashi H, Ochiai H, et al. Fatal degeneration of specialized cardiac muscle associated with chronic active Epstein-Barr virus infection. Pediatr Int. 2009;51:846-8.
2. Guissa VR, Aragão PA, Marques HH, Jacob CM, Silva CA. Chronic active Epstein-Barr virus infection mimicking Henoch-Schönlein purpura. Acta Reumatol Port. 2010;35:513-7.
3. Rodríguez-Pinilla SM, Barrionuevo C, García J, de los Ángeles M, Pajares R, Casavilca S, et al. Epstein-Barr virus-positive systemic NK/T-cell lymphomas in children: report of six cases. Histopathology. 2011;59:1183-93.
4. Hashimoto T, Sakata Y, Fukushima K, Maeda T, Arita Y, Shioyama W, et al. Pulmonary arterial hypertension associated with chronic active Epstein-Barr virus infection. Intern Med. 2011;50:119-24.
5. Wang Y, Xue L, Ma C. Chronic active EBV infection complicated with EBV-related central nervous system T-cell proliferative diseases: report of a case with five-year follow-up. Zhonghua Er Ke Za Zhi. 2011;49:797-800.
6. Lee HY, Baek JO, Lee JR, Park SH, Jeon IS, Roh JY. Atypical hydroa vacciniforme-like epstein-barr virus associated T/NK-cell lymphoproliferative disorder. Am J Dermatopathol. 2012;34:e119-24.
7. Zeng Y, Fu L, Jin H, Sun Q, Wang B. Hydroa vacciniforme-like Epstein-Barr virus-associated lymphoproliferative disease: a case report. Pediatr Dermatol. 2012;29:96-100.
8. Yoshioka K, Fukushima H, Ishii N, Kita A, Hanioka Y, Minami M, et al. A case of chronic active Epstein-Barr virus infection mimicking adult-onset Still's disease. Mod Rheumatol. 2013;23:162-6.
9. Zhang X, Wang Z, Wang L, Yao H. An adult case of systemic Epstein-Barr virus-positive T/natural killer-cell lymphoproliferative disorder with good outcome. Int J Clin Exp Pathol. 2013;6:2620-4.
10. Ichinose K, Origuchi T, Tashiro N, Kawashiri SY, Iwamoto N, Fujikawa K, et al. An elderly patient with chronic active Epstein-Barr virus infection with mixed cryoglobulinemia and review of the literature. Mod Rheumatol. 2013;23:1022-8.
11. Wada Y, Sato C, Tomita K, Ishii-Aso R, Haga H, Okumoto K, et al. Possible autoimmune hepatitis induced after chronic active Epstein-Barr virus infection. Clin J Gastroenterol. 2014;7:58-61.
12. Ameli F, Ghafourian F, Masir N. Systematic Epstein-Barr virus-positive T-cell lymphoproliferative disease presenting as a persistent fever and cough: a case report. J Med Case Rep. 2014;8:288.
13. Kheyri Z, Mojtahedzadeh A, Zamani F, Zaremehrjerdi A, Babaheidarian P. Systemic EBV T-cell lymphoproliferative disease of young adults. Acta Med Iran. 2014;52:286-9.
14. Chen G, Chen L, Qin X, Huang Z, Xie X, Li G, et al. Systemic Epstein-Barr virus positive T-cell lymphoproliferative disease of childhood with hemophagocytic syndrome. Int J Clin Exp Pathol. 2014;7:7110-3.
15. Lemaire AS, Daussay D, Bouchindhomme B, Grardel N, Botte A, Copin MC. Lymphoprolifération systémique T liée à l'EBV chez l'enfant. Ann Pathol. 2014;34:339-43.
16. Jeon YK, Kim JH, Sung JY, Han JH, Ko YH; Hematopathology Study Group of the Korean Society of Pathologists. Epstein-Barr virus-positive nodal T/NK-cell lymphoma: an analysis of 15 cases with distinct clinicopathological features. Hum Pathol. 2015;46:981-90.
17. Xiao HJ, Li J, Song HM, Li ZH, Dong M, Zhou XG. Epstein-Barr Virus-Positive T/NK-Cell Lymphoproliferative Disorders Manifested as Gastrointestinal Perforations and Skin Lesions: A Case Report. Medicine (Baltimore). 2016;95:e2676.
18. Al-Riyami AZ, Al-Farsi K, Al-Khabori M, Al-Huneini M, Al-Hadabbi I. Unusual Indolent Course of a Chronic Active Epstein-Barr Virus-Associated Natural Killer Cell Lymphoproliferative Disorder. Sultan Qaboos Univ Med J. 2016;16:e230-3.
19. Lee JI, Lee SW, Han NI, Ro SM, Noh YS, Jang JW, et al. A Case of Severe Chronic Active Epstein-Barr Virus Infection with Aplastic Anemia and Hepatitis. Korean J Gastroenterol. 2016;67:39-43.
20. Kai K, Koga F, Araki N, Shindo T, Eguchi Y, Toda S, et al. Autopsy case of systemic EBV-positive T-cell lymphoma of childhood with marked hepatomegaly in a middle-aged man. Pathol Int. 2017;67:431-433.
21. Xing Y, Yang J, Lian G, Chen S, Chen L, Li F. Chronic active Epstein-Barr virus infection associated with hemophagocytic syndrome and extra-nodal natural killer/T-cell lymphoma in an 18-year-old girl: A case report. Medicine (Baltimore). 2017;96:e6845.
22. Kaneko H, Taniwaki M, Matsumoto Y, Yoshida M, Shimura K, Fujino T, et al. An adult-onset case of chronic active Epstein-Barr virus infection with fulminant clinical course. J Infect Chemother. 2018;24:479-482.

23. Kawabe A, Nakano K, Miyata H, Shibuya R, Matsuyama A, Ogoshi T, et al. Fatal Chronic Active Epstein-Barr Virus Infection in a Rheumatoid Arthritis Patient Treated with Abatacept. Intern Med. 2019;58:585-591.
24. Wu Q, Ren F, Elston DM. Systemic Epstein-Barr virus-positive T-cell lymphoma of childhood. Cutis. 2019;104:297-300.
25. Wang Z, Zhang Y, Duan M, Zhang Y. Chronic active Epstein-Barr virus infection with intrapulmonary shunting: A case report. J Infect Chemother. 2020;26:502-505.
26. Ondrejka SL, Hsi ED. Chronic active Epstein-Barr virus infection: A heterogeneous entity requiring a high index of suspicion for diagnosis. Int J Lab Hematol. 2020;42 Suppl 1:99-106.
27. Keow JY, Stecho WM, Haig AR, Sangle NA. EBV-positive T/NK-associated lymphoproliferative disorders of childhood: A complete autopsy report. Indian J Pathol Microbiol. 2020;63:78-82.
28. Kwong S, Lu X, Liu X, Lai J. Reactivation of Epstein-Barr Virus Hepatitis in T/Natural Killer (NK) Cells Mimicking Liver T/NK-Cell Lymphoma. Gastroenterology Res. 2020;13:81-84.

List of 8 studies included for evaluating potential selection bias in HSCT patients:

1. Endo T, Mori Y, Fukushi T, Yamaguchi K, Sato K, Sakamoto J, et al. An adult with chronic active Epstein-Barr virus infection associated with repeated liver dysfunction. Nihon Shokakibyo Gakkai Zasshi. 2010;107:1312-8.
2. Tanaka C, Hasegawa M, Fujimoto M, Iwatsuki K, Yamamoto T, Yamada K, et al. Phenotypic analysis in a case of hydroa vacciniforme-like eruptions associated with chronic active Epstein-Barr virus disease of γδ T cells. Br J Dermatol. 2012;166:216-8.
3. Yoshimi Y, Suematsu A, Hisada A, Takamatsu Y, Niwa K, Yokoe M, et al. Case report; A case of chronic active EB virus infection. Nihon Naika Gakkai Zasshi. 2012;101:2298-300.
4. Jeon YK, Kim JH, Sung JY, Han JH, Ko YH; Hematopathology Study Group of the Korean Society of Pathologists. Epstein-Barr virus-positive nodal T/NK-cell lymphoma: an analysis of 15 cases with distinct clinicopathological features. Hum Pathol. 2015;46:981-90.
5. Shimomura M, Morishita H, Meguro T, Seto S, Kimura M, Hamazaki M, et al. Chronic active EBV infection with features of granulomatosis with polyangiitis. Pediatr Int. 2016;58:639-42.
6. Kobayashi N, Mitsui T, Ogawa Y, Iriuchishima H, Takizawa M, Yokohama A, et al. A Rare Case of Chronic Active Epstein-Barr Virus (EBV) Infection Accompanied by the Infiltration of EBV-infected CD8+ T Cells into the Muscle. J Pediatr Hematol Oncol. 2018;40:e171-e175.
7. Tanita K, Hoshino A, Imadome KI, Kamiya T, Inoue K, Okano T, et al. Epstein-Barr Virus-Associated γδ T-Cell Lymphoproliferative Disorder Associated With Hypomorphic IL2RG Mutation. Front Pediatr. 2019;7:15.
8. Hung GY, Yu TY, Yen HJ, Yang CF, Lin LY, Horng JL. Systemic Epstein-Barr Virus-positive T-Cell Lymphoma of Childhood Presentation With Hemophagocytosis. J Pediatr Hematol Oncol. 2019;41:319-320.
